# Prognostic impact of oral anticoagulation therapy and atrial fibrillation in patients with type B acute aortic dissection

**DOI:** 10.1002/joa3.13020

**Published:** 2024-03-07

**Authors:** Naruya Ishizue, Hidehira Fukaya, Jun Oikawa, Nobuhiro Sato, Sho Ogiso, Yusuke Murayama, Hironori Nakamura, Jun Kishihara, Shinichi Niwano, Junya Ako

**Affiliations:** ^1^ Department of Cardiovascular Medicine Kitasato University School of Medicine Sagamihara Japan; ^2^ Department of Kitasato Clinical Research Center Kitasato University School of Medicine Sagamihara Japan

**Keywords:** acute aortic dissection, atrial fibrillation, cardiovascular event, oral anticoagulation therapy

## Abstract

**Background:**

The prognostic impact of atrial fibrillation (AF) and oral anticoagulation (OAC) therapy in patients with type B acute aortic dissection (AAD) remains unclear. Therefore, we investigated the prognostic impact of AF and OAC therapy in patients with type B AAD.

**Methods:**

Consecutive patients diagnosed with AAD were included in this single‐center, retrospective study. Patients with type B AAD were selected from the study population and divided into three groups: AF(+)/OAC(+), AF(+)/OAC(−), and AF(−)/OAC(−). The primary end point was major adverse cardiovascular and cerebrovascular events (MACCE), including all‐cause death, progressive aortic events, cerebral infarction, and organ malperfusion.

**Results:**

In total, 139 patients diagnosed with type B AAD were analyzed. AF was observed in 27 patients (19%). Among them, 13 patients (9%) received OAC therapy for AF. MACCE occurred in 32 patients (23%) during the observation period: all‐cause death in four patients, progressive aortic events in 24 patients, cerebral infarction events in two patients, and malperfusion events in two patients. The incidence of MACCE was higher in the AF(+)/OAC(+) group than in the AF(+)/OAC(−) group (hazard ratio[HR]: 3.875; 95% confidence interval [CI]: 1.153–17.496). In contrast, there was no significant difference in the incidence of MACCE between the AF(+)/OAC(−) and AF(−)/OAC(−) groups (HR: 1.001, 95% CI: 0.509–1.802).

**Conclusion:**

Among patients with type B AAD, the use of OAC for AF was associated with a higher risk of MACCE.

## Graphical abstract

The prognostic impact of atrial fibrillation (AF) and oral anticoagulation (OAC) therapy in patients with type B acute aortic dissection (AAD) remains unclear. The use of OAC for AF was related to poor prognosis in patients with type B AAD.

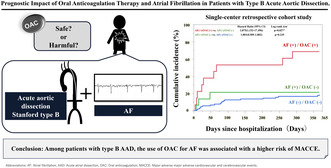

## 1 INTRODUCTION

Acute aortic dissection (AAD) is often complicated by atrial fibrillation (AF), with an incidence of 12%–17%.^1^, ^2^, ^3^ These conditions share common risk factors, including atherosclerosis, hypertension, sleep apnea, and advanced age.^4^, ^5^, ^6^, ^7^, ^8^, ^9^, ^10^ However, research on whether the presence of AF contributes to a worse prognosis in patients with AAD is lacking. Furthermore, despite the importance of oral anticoagulation (OAC) therapy for preventing cardiogenic thromboembolism in patients with AF, there are no specific recommendations for OAC management in patients with AAD and AF. Although there are several reports on the safety of OAC in preventing thromboembolism in patients with AAD,^1^, ^2^, ^3^, ^4^, ^5^, ^6^, ^7^, ^8^, ^9^, ^10^, ^11^, ^12^, ^13^ comprehensive data on thromboembolic events and the risk of worsening dissection are limited. Therefore, this study aims to assess the prognostic impact of AF and OAC therapy in patients with AAD.

## 2 MATERIALS AND METHODS

### 2.1 Study population and diagnosis criteria

**FIGURE 1:**
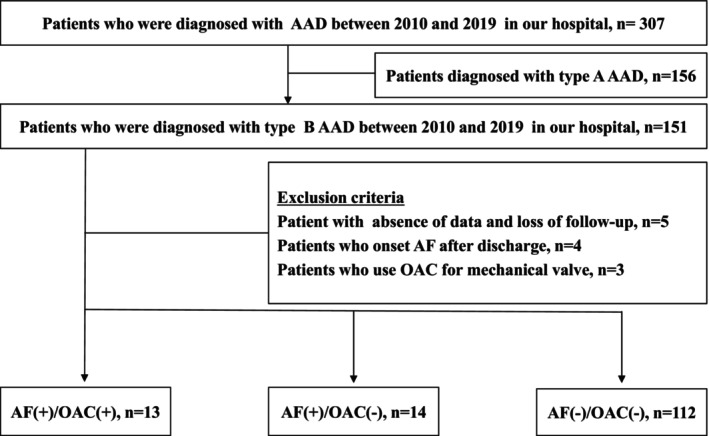
Study population. In total, 307 patients diagnosed with AAD were enrolled in this study. Among them,139 patients diagnosed with type B AAD were analyzed. The patients were divided into three groups based on the presence or absence of AF and the use of OAC: AF(+)/OAC(+), AF(+)/OAC(−), and AF(−)/OAC(−). AAD, acute aortic dissection; AF, atrial fibrillation; OAC, oral anticoagulation.

We performed a retrospective analysis of consecutive patients who were diagnosed with AAD at Kitasato University Hospital between 2010 and 2019. Since the prognosis and indication for surgery for AAD differed between Stanford classification types A and B, patients with type A AAD were excluded, and only patients diagnosed with type B AAD were included in this study. Additionally, patients with missing data (follow‐up data, history, and medication history), patients with AF onset after discharge, and those receiving OAC for mechanical valves were excluded (Figure 1).

Type B AAD was defined as any nontraumatic dissection involving the descending aorta that appeared within 14 days of symptom onset.^14^, ^15^, ^16^ The diagnosis was based on computed tomography (CT) findings, which confirmed a dissected descending aorta. Based on clinical features, all cases were classified into the nonthrombosed type, which was characterized by partial enhancement of the false lumen; and the thrombosed type, which was characterized by a false lumen without enhancement, including an intramural hematoma of the aorta.^16^

AF was defined as an irregular ventricular rate and the absence of P waves on a standard 12‐leads electrocardiogram (ECG) or lasting >30 s on a continuously monitored ECG. In this study, AF was defined as the diagnosis of AF during or before hospitalization. Patients with new‐onset AF after hospital discharge were excluded. The types of AF were classified according to their temporal patterns: paroxysmal or nonparoxysmal AF. Paroxysmal AF was defined as spontaneous termination within 7 days.^17^ All other patients were defined as having nonparoxysmal AF. New‐onset AF was defined as newly detected AF during hospitalization without a history of AF. The indication of OAC therapy was at the discretion of the physician according to the guideline recommendations.^18^ OAC use was defined as an oral prescription during the hospital stay or prior to admission. All patients were divided into three groups based on the presence or absence of AF and the use of OAC: AF(+)/OAC(+), AF(+)/OAC(−), and AF(−)/OAC(−).

### 2.2 Baseline characteristics and clinical outcomes

Baseline patient characteristics were retrospectively assessed, including age, gender, body mass index, medical history, laboratory data, imaging results, and electrocardiograms. The primary end point was defined as major adverse cardiovascular and cerebrovascular events (MACCE), which included all‐cause death, progressive aortic events, organ malperfusion, and cerebral infarction during the observation period. Progressive aortic events were defined as aortic rupture, enlargement of the aortic dimension (>60 mm), recurrence of dissection, and early aortic expansion (≥1 mm/day), which were indications for surgical treatment.^14^, ^19^ These events were diagnosed by CT imaging. The day of AAD onset was estimated based on the symptoms related to AAD, or the first day on which AAD was diagnosed using CT if the symptoms were unclear.

After the initial event, patients underwent CT imaging 48 h after onset, and follow‐up CT scans were performed at 6 months and 1 year after onset. Alternatively, additional CT scans were performed according to subjective symptoms and other findings of concern. The use of contrast media was not mandatory for any CT scan. The observational period was set at 1 year after AAD onset.

### 2.3 Statistical analysis

The statistical analyses were conducted using the JMP® version 11.2 statistical software package (SAS Institute Inc., Cary, NC, USA). Baseline characteristics are presented as medians and interquartile ranges for continuous data and as percentages for categorical data. Continuous variables were compared using either the Mann–Whitney *U* or the Steel–Dwass test, while categorical variables were assessed through the chi‐square test or Fisher's exact test. Event‐free rates were assessed using Kaplan–Meier survival analysis with the log‐rank test. Cox proportional hazards regression analysis was utilized to investigate the adjusted effects of risk factors on survival. The threshold for statistical significance was set at *p* < .05.

## 3 RESULTS

### 3.1 Study flowchart and comparison of patients' characteristics

In total, 307 patients diagnosed with AAD were enrolled in this study. One hundred fifty‐one patients were diagnosed with type B AAD. Five patients were excluded because of the absence of data or loss of follow‐up. Four patients with AF onset after discharge and three who underwent OAC for mechanical valves were excluded (Figure 1). Consequently, 139 patients were analyzed, with a median age of 68 [58–76] years, and 76% were male. According to the subtypes of type B AAD, 46 patients (33%) had nonthrombosed AAD, and 93 (67%) had thrombosed AAD, including an intramural hematoma of the aorta. The median diameter of the maximum descending aorta at admission was 35 [32֪–40] mm. The patients were treated with calcium channel blockers (82%), angiotensin‐converting enzyme inhibitors or angiotensin receptor blockers (80%), β blockers (94%), and α‐blockers (19%). AF was observed in 27 (19%) patients. Among them, 13 patients (9%) received OAC therapy for AF: two patients (1%) received warfarin, and 11 patients (8%) received direct oral anticoagulants (DOACs) (Table 1). All patients were classified into the following three groups: AF(+)/OAC(+): 13 patients, AF(+)/OAC(−): 14 patients, and AF(−)/OAC(−): 112 patients. The CHADS_2_ score was significantly higher in the AF(+)/OAC (+) group than in the other groups (CHADS_2_ score, AF(+)/OAC (+): 1 [1, 2] vs. AF(+)/OAC (−): 0 [0–1] vs. AF(−)/OAC(−): 0 [0–1], *p* = .049). Although not statistically significant, there was a trend toward more preexisting hypertension in the AF (+)/OAC (+) group than in the other groups (Table 1).

**TABLE 1:** Comparison of baseline characteristics between each group.

	Total	AF(+)/OAC(+)	AF(+)/OAC(−)	AF(−)/OAC(−)	*p*‐Value
	*n* = 139	*n* = 13	*n*‐14	*n* = 112	
Demographic data					
Male, *n* (%)	105 (76)	13 (100)	11 (79)	81 (72)	.094
Age (years old)	68 [58–76]	77 [70–80]	67 [52–72]	66 [56–76]	.054
Body mass index (kg/m^2^)	22 [20–24]	22 [19–25]	20 (20–24)	23 [21–28]	.302
Underlying disease					
Hypertension, *n* (%)	81 (58)	10 (77)	6 (43)	65 (58)	.198
Diabetes mellitus, *n* (%)	11 (8)	0 (0)	1 (7)	10 (90)	.523
Dyslipidemia, *n* (%)	22 (16)	1 (8)	0 (0)	21 (19)	.137
Stroke, *n* (%)	10 (7)	1 (8)	0(0)	9 (8)	.546
Congestive heart failure, *n* (%)	3 (2)	1 (8)	1 (7)	1 (1)	.112
Vascular disease, *n* (%)	19 (14)	3 (23)	2 (14)	14 (12)	.809
CHADS_2_ score	1 [0–1]	1 [1–2]	0 [0–1]	0 [0–1]	.049*
CHA_2_DS_2_‐VASc score	2 [1–3]	3 [2–3]	2 [1–2]	2 [1–3]	.351
AF type					
Paroxysmal AF, *n* (%)	24 (17)	10 (77)	14 (100)	NA	.057
Nonparoxysmal AF, *n* (%)	3 (2)	3 (23)	0 (0)	NA	–
New onset AF, *n* (%)	11 (8)	4 (31)	7 (50)	NA	.309
Preexisting AF, *n* (%)	16 (12)	9 (69)	7 (50)	NA	–
Clinical presentation on admission					
Heart rate (bpm)	78 [66–92]	76 [71–115]	70 [64–92]	80 [66–106]	.843
Systolic blood pressure (mmHg)	165 [132–204]	154 [124–215]	132 [119–215]	166 [138–231]	.554
Laboratory data at discharge					
Cr (mg/dL)	0.91 [0.72–1.07]	1.01 [0.92–1.07]	1.04 [0.82–1.19]	9.86 [0.70–1.60]	.848
CRP (mg/L)	0.11 [0.36–1.29]	1.06 [0.36–2.00]	0.36 [0.13–1.13]	0.26 [0.10–1.29]	.267
D‐Dimmer (μg/mL)	5.71 [2.73–13.07]	3.73 [1.97–5.97]	5.94 [2.62–14.93]	5.77 [2.79–15.47]	.389
Echocardiogram parameter					
Ejection fraction (%)	65 [61–70]	61 [55–64]	65 [57–70]	67 [62–71]	.001
Left atrial diameter (mm)	37 [32–40]	40 [37–44]	38 [33–40]	36 [32–40]	.022*
Initial CT findings					
Maximum ascending aorta diameter (mm)	37 [34–37]	39 [35–45]	37 [34–40]	37 [34–39]	.164
Maximum descending aorta diameter (mm)	35 [32–40]	48 [33–43]	37 [34–42]	35 [30–39]	.421
Maximum false lumen diameter (mm)	14 [10–19]	12 [9–18]	14 [10–20]	14 [10–18]	.494
Nonthrombosed type, *n* (%)	46 (33)	5 (38)	4 (29)	37 (26)	.862
Oral medication during hospitalization					
ACEi/ARB, *n* (%)	111 (80)	9 (69)	9 (64)	93 (84)	.185
Ca blocker, *n* (%)	114 (82)	11 (84)	10 (71)	93 (67)	.582
β blocker, *n* (%)	122 (94)	13 (100)	13 (93)	106 (76)	.656
α blocker, *n* (%)	27 (19)	1 (8)	1 (7)	25 (18)	.213
OAC therapy, *n* (%)	13 (9)	–	–	–	–
Warfarin, *n* (%)	2 (1)	2 (15)	NA	NA	NA
Direct oral anticoagulant, *n* (%)	11 (8)	11(85)	NA	NA	NA

Abbreviations: ACEi/ARB, angiotensin‐converting enzyme inhibitor/angiotensin II receptor blocker; AF, atrial fibrillation; BMI, body mass index; Cr, creatinine; CRP, C‐reactive peptide; OAC, oral anticoagulantion.

* Asterisk indicates significance.

### 3.2 Clinical predictor for MACCE at 1 year

In univariate analysis, MACCE were associated with age >70 years (hazard ratio [HR]: 3.468, 95% confidence interval [CI]: 1.657–7.921, *p* = .001), complications with AF (HR: 3.080, 95% CI: 1.462–6.229, *p* = .004), use of OAC (HR: 5.273, 95% CI: 2.304–11.059, *p* = .001), and maximum descending aorta diameter (per 1 mm, HR: 1.059, 95% CI: 1.013–1.102, *p* = .012) (Table 2).

**TABLE 2:** Risk factor for MACCE.

Variables	Univariate analysis		
	HR	95% CI	*p*‐Value
Age >70 years	3.468	1.657–7.921	.001*
Male	1.467	0.254‐1.548	.379
Hypertension	1.031	0.513–2.136	.933
Diabetes mellitus	0.732	0.112–2.425	.692
Dyslipidemia	1.217	0.453–2.766	.671
Stroke	0.37	0.021–1.724	.25
Congestive heart failure	3.346	0.861–10.712	.07
Heart rate	0.994	0.974–1.013	.525
Systolic blood pressure	0.997	0.989–1.005	.453
Cr	0.971	0.699–1.167	.793
D‐Dimmer	0.998	0.976–1.008	.771
CRP	1.027	0.938–1.102	.525
AF	3.08	1.462–6.229	.004*
CHADS_2_ score	1.255	0.899–1.689	.173
OAC	5.273	2.304–11.059	.001*
Ejection fraction	0.985	0.948–1.038	.498
Left atrial diameter	1.001	0.968–1.109	.781
Maximum descending aorta diameter	1.059	1.013–1.102	.012*
False lumen diameter	1.033	0.963–1.074	.497
Nonthrombosed type	1.019	0.474–2.073	.959

Abbreviations: AF, atrial fibrillation; CI, confidence interval; Cr, creatinine; CRP, C‐reactive peptide; HR, hazard ratio; OAC, oral anticoagulation.

* Asterisk indicates significance.

### 3.3 Clinical outcomes of patients with type B AAD during the 1‐year observation

The MACCE was observed in 32 patients: all‐cause death in four patients, progressive aortic events in 24 patients, cerebral infarction events in two patients, and malperfusion events in two patients. Of the 24 patients with progressive aortic events, aortic rupture occurred in seven (29%), dissection recurred in three (13%), enlargement of the aortic dimension occurred in 10 (42%), and early aortic expansion occurred in four (17%) (Figure S1).

The incidence of MACCE was higher in the AF(+)/OAC(+) group than in the AF(+)/OAC(−) group (HR: 3.875, 95% CI: 1.153–17.496, *p* = .028; Table 3). The incidence of progressive aortic events was higher in the AF(+)/OAC(+) group than in the AF(+)/OAC(−) group (HR: 4.664, 95% CI: 1.123–31.358, *p* = .033; Table 3). Notably, MACCE often occurred within 100 days of admission. However, there was no significant difference in the incidence of MACCE between the AF(+)/OAC(−) and AF(−)/OAC(−) groups (HR: 1.001, 95% CI: 0.509–1.802, *p* = .977; Table 3).

**TABLE 3:** Primary outcomes among each group.

Variables	Total	AF(+)/OAC(+)	AF(+)/OAC(−)	AF(−)/OAC(−)	AF(+)/OAC(−) versus AF(−)/OAC(−)		AF(+)/OAC(+) versus AF(+)/OAC(−)	
	*n* = 139	*n* = 13	*n* = 14	*n* = 112	Hazard ratio (95% CI)	*p*‐Value	Hazard ratio (95% CI)	*p*‐Value
MACCE, *n* (%)	32 (23)	9 (69)	3 (21)	20 (18)	1.001 (0.509–1.802)	.977	3.875 (1.153–17.496)	.028*
All cause death, *n* (%)	4 (3)	0 (0)	0 (0)	4 (4)	0.904 (0.537–1.649)	.727	NA	NA
Progressive aortic event, *n* (%)	24 (17)	7 (54)	2 (14)	15 (13)	1.046 (0.545–1.827)	.884	4.664 (1.123–31.358)	.033*
Cerebral infarction, *n* (%)	2 (1)	2 (15)	0 (0)	0 (0)	NA	NA	1.381 (0.608–3.057)	.431
Malperfusion, *n* (%)	2 (1)	0 (0)	1 (7)	1 (1)	1.000 (0.585–1.865)	.999	0.582 (0.265–1.278)	.175

Abbreviation: MACCE, major adverse major adverse cardiovascular and cerebrovascular events.

* Asterisk indicates significance.

An adjusted analysis using factors obtained from the univariate analysis, such as maximum descending aortic diameter and age, was performed in addition to AF and OAC. After adjustment, the incidence of MACCE was higher in the AF(+)/OAC(+) group than in the AF(+)/OAC(−) group (HR: 4.130, 95% CI: 1.010–21.831, *p* = .0483; Table 4).

**TABLE 4:** Primary outcomes adjusted by each parameter.

Adjusted by age, maximum descending aorta diameter		
Variables	AF(+)/OAC(+) versus AF(+)/OAC(−)	
	HR (95% CI)	*p*‐Value
MACCE	4.130 (1.010–21.831)	.048*
All‐cause death	NA	NA
Progressive aortic event	4.971 (0.960–39.348)	.056
Cerebral infarction	1.603 (0.544–4.382)	.391
Malperfusion	0.553 (0.190–1.548)	.261

Abbreviation: MACCE, major adverse major adverse cardiovascular and cerebrovascular events.

* Asterisk indicates significance.

**FIGURE 2:**
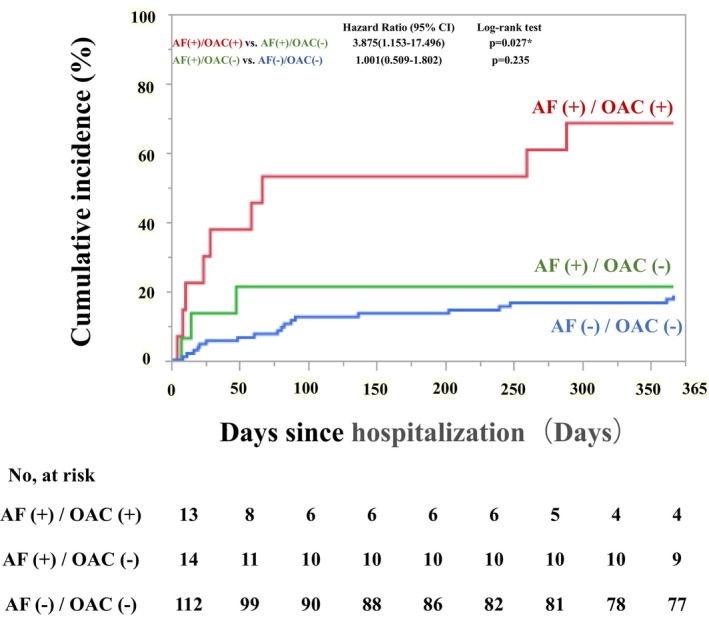
Kaplan–Meier curves comparing MACCE in each group. The Kaplan–Meier curves of freedom from the MACCE in each group. Patients with AF(+)/OAC(+) had a significantly higher incidence of MACCE than patients with AF(+)/OAC(−) (log‐rank *p* = .027). However, The incidence of MACCE was not significantly different between AF(+)/OAC(−) and AF(−)/OAC(−) groups (log‐rank *p* = .285). AF, atrial fibrillation; MACCE, major adverse cardiovascular and cerebrovascular events; OAC, oral anticoagulation.

Figure 2 shows the Kaplan–Meier curves of freedom from the MACCE in each group. Patients with AF(+)/OAC(+) had a significantly higher incidence of MACCE than patients with AF(+)/OAC(−) (log‐rank *p* = .027). However, The incidence of MACCE was not significantly different between AF(+)/OAC(−) and AF(−)/OAC(−) groups. (log‐rank *p* = .285).

**FIGURE 3:**
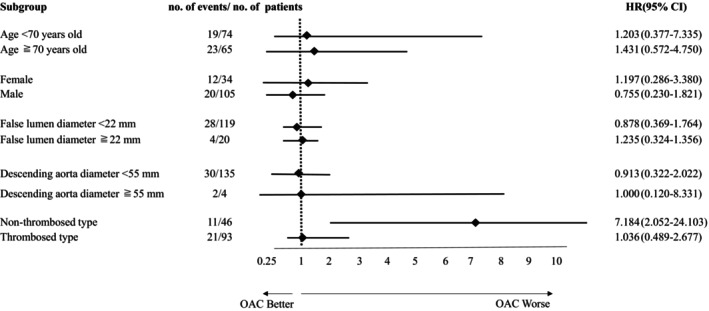
Subgroup analysis of MACCE. Hazard ratios were calculated using the Cox logistic regression analysis. OAC use was associated with a higher risk in nonthrombosed patients than in thrombosed patients. 95% CI, 95% confidence interval; AF, atrial fibrillation; HR, hazard ratio; MACCE, major adverse cardiovascular and cerebrovascular events; OAC, oral anticoagulation.

In the subgroup analysis, OAC use for AF had a poor prognosis in the patients with the nonthrombosed type (HR: 7.184, 95% CI: 2.052–24.103; Figure 3).

## 4 DISCUSSION

In this single‐center retrospective cohort study, we evaluated the prevalence of AF in patients with type B AAD and the impact of AF and OAC treatment on MACCE in these patients. Our findings are as follows: Out of the 139 patients diagnosed with Stanford type B AAD, 27 (19%) exhibited AF, and 32 (23%) experienced MACCE during the 1‐year observation. Notably, AF itself could not be an independent predictor of MACCE. However, it is important to highlight that the incidence of MACCE was elevated among patients who were prescribed OAC for AF.

### 4.1 Prognostic impact of AF for AAD

The incidence of AF in AAD patients is reported to be approximately 12%–17%.^1^, ^2^, ^3^ In this study, AF was observed in 27/139 patients (19%), similar to the findings of previous studies. A study based on a Taiwanese national database reported that patients with AF had a 1.18‐fold higher incidence of AAD than those without AF.^2^ Nevertheless, patients with type B AAD generally have a better prognosis than patients with type A AAD. However, the International Registry of Acute Aortic Dissection (IRAD) reported a 3‐year survival rate of 78% in patients with type B AAD treated with conservative management.^20^ In other words, cardiovascular or fatal events occurred in 22% of patients, even in those with type B AAD; advanced age, female, renal dysfunction, initial aortic enlargement, and high C‐reactive protein levels are poor prognostic factors in such patients.^1^, ^21^, ^22^, ^23^, ^24^ There is a lack of data regarding whether the presence of AF contributes to a worsening prognosis in patients with type B AAD. Campia et al. reported that AF is a poor prognostic event in patients with acute aortic syndrome.^25^ In this report, 60% of patients with AF were prescribed OAC; however, the impact of OAC use was not addressed in the analysis. In this study, univariate analysis revealed that AF, OAC, age, and maximum descending aortic diameter were prognostic factors for MACCE. OAC was prescribed to 48% of the patients with AF in this cohort. Thus, it can be extrapolated that the presence of AF and the use of OAC may have an interaction effect. Additionally, we could not perform multivariate analysis, because of the small sample size. This study found no significant difference in MACCE between the AF(+)/OAC(−) and AF(−)/OAC(−) groups. Therefore, we concluded that AF itself could not be an independent predictor of MACCE. Interestingly, factors associated with the incidence of AF—age, hypertension, and diabetes mellitus—overlap with factors of atherosclerosis progression. It may suggest that the presence of AF is a phenotype and not an exacerbation factor of advanced atherosclerosis or AAD progression.

### 4.2 Effect of OAC therapy for AF in patients with AAD

As shown in Table 1, patients with nonparoxysmal AF received OAC therapy. Although not statistically significant, there was a trend toward more preexisting hypertension in the AF (+)/OAC (+) group than in the other groups. Table S1 shows the details of the patients taking OAC. Three of the patients had no history of hypertension. The CHADS_2_ score was ≥1 for all patients taking OAC. Low‐risk patients were not prescribed OAC. Although the CHADS_2_ score was higher in the AF(+)/OAC(+) group than in the other groups, the use of OAC was determined a risk factor for MACCE in the analysis adjusted only for the CHADS_2_ score (data not shown). In this study, the incidence of MACCE was associated with the use of OAC for AF. However, several investigators have reported the safety of OAC therapy in patients with AAD.^10^, ^11^, ^12^, ^13^ The efficacy of OAC therapy in patients with type B AAD and AF remains controversial.

AF is a potent risk factor for cardiogenic thromboembolism. High CHADS_2_/CHA_2_DS_2_‐VASc scores indicate the risk of cardiogenic thromboembolism. Current guidelines recommend OAC therapy to prevent stroke and systemic thromboembolism based on the CHADS_2_/CHA_2_DS_2_‐VASc score.^18^, ^26^ Vascular disease is one of the factors constituting the CHA_2_DS_2_‐VASc score, and patients with AAD often have hypertension; therefore, the CHA_2_DS_2_‐VASc score of patients with AAD and AF would be >2, resulting in these patients potentially being indicated for anticoagulation. However, the guidelines do not explicitly mention OAC therapy for patients with AAD and AF.^18^, ^26^ Moreover, a previous cohort study revealed that warfarin anticoagulation therapy was not associated with late mortality or late aortic events in patients with postsurgical type A AAD.^27^ However, clinical studies on the use of OAC for treating AF in patients with type B AAD are scarce. In this study, OAC use for AF was associated with a higher incidence of MACCE. Notably, MACCE often occurred within 100 days of admission in this study. Based on these results, indications for OAC therapy during the acute and subacute phases in patients with AAD and AF should be carefully considered. This study could not conclude that the use of OACs in patients with AAD and AF should be avoided because of the small sample size from a single center. At present, the indications for OAC therapy in patients with AAD and AF should be discussed individually.

Additionally, the subgroup analysis suggested that the prevalence of the nonthrombosed type could be a worsening factor in patients using OAC (Figure 3). Of the 13 patients on OAC, nine experienced a MACCE event. Furthermore, five patients on OAC with MACCE events were nonthrombosed: they had a false lumen communicating with the true lumen on follow‐up CT scans (Table S1). A previous report showed that residual false lumen blood flow is a prognostic factor for AAD.^28^ These results suggest that the use of OAC disturbs thrombotic formation in the false lumen during the acute phase. The use of OAC may be a clinical dilemma for patients with AAD and AF who have a high risk of thrombosis. Concerning this issue, the left atrial appendage exclusion using an epicardial clip device may be suitable for avoiding OAC therapy for AAD patients with high CHA_2_DS_2_‐VASc score.^29^ Moreover, since thoracic endovascular aortic repair was effective in patients with chronic aortic dissection in the INSTEAD XT trial,^30^ it would also apply to patients with chronic nonthrombosed type aortic dissection.

## 5 LIMITATIONS

This study had several limitations. First, the study population was small as this was a single‐center clinical study. Moreover, the observational period was limited to 1 year; therefore, the long‐term outcomes remain unclear. Second, co‐occurrence with AF was defined as the presence of AF on admission or during hospitalization. We did not evaluate patients who developed AF after discharge. Therefore, the impact of AF and OAC use on aortic dissection in the chronic phase has not been assessed. Third, the indication for OAC use was based on the CHADS_2_ score, but the attending physician made the final decision. In addition, patients receiving OACs may have more regular outpatient visits. These factors might have introduced a selection bias. Fourth, it was not possible to determine which type of patients with AF and aortic dissection should be prescribed OACs. Finally, we could not elucidate the detailed mechanisms of MACCE caused by OAC use. Further studies are needed to evaluate the impact of OAC therapy on MACCE in patients with type B AAD in a larger population.

## 6 CONCLUSIONS

The use of OAC for AF was associated with a higher risk of MACCE in patients with type B AAD and AF. These findings highlight the contentious indications for OAC therapy in patients with type B AAD and AF. OAC therapy for AF should be considered with caution in patients with type B AAD, especially in those with nonthrombosed AAD during the acute and subacute phases.

## FUNDING INFORMATION

None.

## CONFLICT OF INTEREST STATEMENT

The authors declare no conflicts of interest. No unapproved use of compounds or products was observed.

## ETHICS STATEMENT

This study was approved by the Clinical Studies and Ethics Committee of Kitasato University Hospital (reference number: B21‐096).

## PATIENT CONSENT STATEMENT

Informed consent was obtained in the form of opt‐out.

## PERMISSION TO REPRODUCE MATERIAL FROM OTHER SOURCES

This article does not include reproducible materials from other sources.

## CLINICAL TRIAL REGISTRATION

None.

## Supporting information


Figure S1:

Table S1:

## Data Availability

Deidentified participants' data will be shared on a request basis. Please contact the corresponding author directly to request data sharing.
