# Supplementary material for: Prognostic impact of oral anticoagulation therapy and atrial fibrillation in patients with type B acute aortic dissection
Source: J Arrhythm. 2024 Mar 7;40(2):297–305. doi: 10.1002/joa3.13020 (PMC10995604; doi:10.1002/joa3.13020)
Supplement: Supplementary file 1 — Figure S1: [file JOA3-40-297-s001.pptx]

## Slide 1
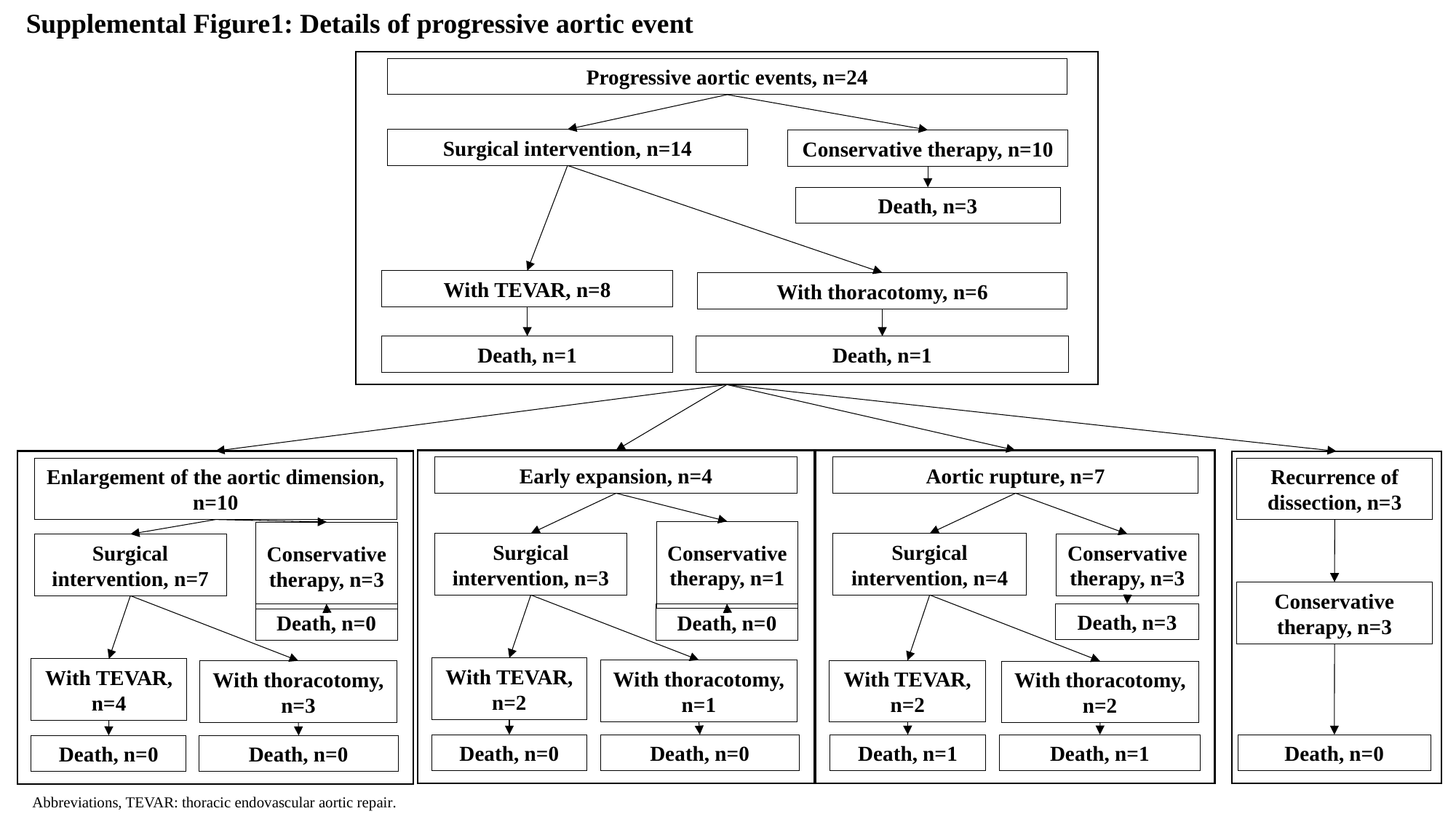

Supplemental Figure1: Details of progressive aortic event
Progressive aortic events, n=24
Surgical intervention, n=14
Conservative therapy, n=10
Death, n=3
With TEVAR, n=8
With thoracotomy, n=6
Death, n=1
Death, n=1
Early expansion, n=4
Surgical intervention, n=3
Conservative therapy, n=1
Death, n=0
With TEVAR, n=2
With thoracotomy, n=1
Death, n=0
Death, n=0
Aortic rupture, n=7
Surgical intervention, n=4
Conservative therapy, n=3
Death, n=3
With TEVAR, n=2
With thoracotomy, n=2
Death, n=1
Death, n=1
Enlargement of the aortic dimension, n=10
Surgical intervention, n=7
Conservative therapy, n=3
Death, n=0
With TEVAR, n=4
With thoracotomy, n=3
Death, n=0
Death, n=0
Recurrence of dissection, n=3
Conservative therapy, n=3
Death, n=0
Abbreviations, TEVAR: thoracic endovascular aortic repair.
